# Nutritional management of lactose intolerance: the importance of diet and food labelling

**DOI:** 10.1186/s12967-020-02429-2

**Published:** 2020-06-26

**Authors:** Maria Sole Facioni, Benedetta Raspini, Francesca Pivari, Elena Dogliotti, Hellas Cena

**Affiliations:** 1 AILI - Associazione Italiana Latto-Intolleranti Onlus, Lucca, Italy; 2 Dietetics and Clinical Nutrition Laboratory, Department of Public Health, Experimental and Forensic Medicine, University of Pavia, Pavia, Italy; 3 Biochemistry and Molecular Biology Laboratory, Department of Health Sciences, University of Milan, Milan, Italy; 4 Fondazione Umberto Veronesi, Milan, Italy; 5 Clinical Nutrition and Dietetics Service, Unit of Internal Medicine and Endocrinology, ICS Maugeri IRCCS, Pavia, Italy

**Keywords:** Lactose intolerance, Nutritional approach, Lactose-free labelling, Nutritional deficiency, Food hidden lactose, Lactose food content, Lactose intolerance management

## Abstract

Worldwide, 70% of the adult population has limited expression of lactase enzyme with a wide variation among different regions and countries. Lactase deficiency may lead to lactose intolerance (LI). Depending both on the amount of lactose ingested and on the lactase activity, people who suffer from lactose malabsorption might experience numerous gastrointestinal and extra-intestinal symptoms and manifestations. Treatment of LI mainly consists of reducing or eliminating lactose from the diet until the symptoms disappear as well as supplementing lactase, and inducing colon microbiome adaptation by probiotics. Cow’s milk is one of the major source of calcium and several other vitamins and minerals. Thus, a complete exclusion of dairy products may favor the development of bone diseases such as osteopenia and osteoporosis. Therefore, the dietetic approach has a crucial role in the management of LI patients. Additionally, the use of lactose and milk-derived products in non-dairy products (e.g., baked goods, breakfast cereals, drinks, and processed meat) has become widespread in the modern industry (the so-called “hidden lactose”). In this regard, a strict adherence to the lactose-free diet becomes challenging for LI patients, forced to continuous check of all products and food labels. In fact, lactose-free product labeling is still controversial. Considering that nowadays a specific cut-off value establishing “lactose-free” labeling policy is lacking and that there is no universal law regulating the production and commercialization of “delactosed” products, identification of specific safe and suitable products with a well-recognized lactose-free logo might help consumers. This narrative review aims to identify the dietary management for lactose intolerant people, avoiding symptoms and nutrients deficiencies, helped by the use of specific labelling to guide them to choose the safer product on the market.

## Background

Lactose intolerance (LI) occurs when the small intestine does not produce enough of the lactase enzyme to digest lactose, the sugar found in milk [1].

There is significant ambiguity regarding the terms used to define lactose metabolism that are often confused [2]. Lactose malabsorption (LM) occurs when non-digested lactose passes through the gut without being absorbed. The undigested lactose in the lumen becomes subject to bacterial fermentation, increasing the osmotic load and resulting in intolerance symptoms after lactose ingestion [3, 4]. When LM is coupled with the above symptoms, it is usually referred to as LI [2, 3]. Depending on both on the amount of lactose ingested and on the activity of the lactase, people who suffer from LM might experience numerous gastrointestinal symptoms, (e.g., abdominal pain, bloating, borborygmi, nausea, diarrhea and/or constipation) and extraintestinal symptoms and manifestations (e.g., headache, severe fatigue, cognitive dysfunction, muscle and/or joint pain, skin lesions, mouth ulcers, heart palpitations, eczema, urticarial and increased micturition). On the contrary, others might have no symptoms after ingestion of a standard serving of dairy products: 125 g for milk and yogurt, 100 g for fresh cheeses and 50 g for hard and aged cheeses [2, 3, 5–8].

Approximately, 70% of the adult population worldwide has a limited expression of lactase enzyme, with a wide variation between different regions and countries [4, 9, 10]. This condition occurs for one of two reasons: genetically determined lactase non-persistence [11] or the presence of another gastrointestinal disorders. In both cases, this could lead to LM and LI [12].

Nowadays, the gold standard methodology for determining LI is the H_2_/CH_4_ Lactose Breath Test (LBT) coupled with the genetic test evaluating lactase-gene polymorphism [13]. However, diagnosis remains challenging, and the proper interpretation of different tests is necessary to identify the most appropriate therapeutic strategy [13–17].

The treatment for LI consists mainly of reducing or eliminating lactose from the diet until the symptoms disappear. Therefore, the dietetic approach has a crucial role in the management of LI patients.

The right approach includes a lactose-free, or low-lactose diet, oral lactase enzyme replacement, and colon microbiome adaptation, using specific probiotic strains with β-galactosidase enzymatic activity [7].

Lactose is commonly found in dairy products, such as milk, yogurt, cream, butter, ice cream, and cheese. However, lactose can also be found in some bread and baked foods, ready-to-eat breakfast cereals, instant soups, confectionery, biscuits, salad dressings, sausages, gravy, drink mixes, and margarine: the so-called “hidden lactose”. Additionally, lactose can also be hidden in prescription and over-the-counter medications [7].

Considering that a lactose-free diet is a key treatment for patients diagnosed with LI, it is essential for affected individuals to stay away from selected dairy products and non-dairy foods that contain milk and/or lactose ingredients. Therefore, it is important to pay careful attention to the ingredients lists of products being consumed. Words that indicate the presence of lactose include curds, whey, milk, milk by-products, dry milk solids, and milk powder.

Fortunately, individuals with LI, do not need to completely eliminate dairy products from their diet. In today’s market, there are excellent solutions for LI people. These include naturally lactose-free, and the development of products that rely on the hydrolysis of lactose, into glucose and galactose, using the enzyme lactase.

The aim of this narrative review is to identify the best dietetic strategy for lactose intolerant individuals, to avoid symptoms and nutrient deficiency (e.g. calcium), helped by the use of specific labelling to guide them to choose the safer product on the market.

## Nutritional management in LI patients

Individuals with LI are usually instructed to follow a lactose-free diet to reduce symptom manifestations [7]. However, the avoidance of all dairy products in patients with LI is no longer recommended today, as the majority of LI patients can tolerate up to 5 g of lactose per single dose—approximately the equivalent of 100 mL of milk. The tolerance threshold increases if the lactose is consumed together with other nutrients. In this context, it would be useful to have an authoritative guide on which products to choose in order to not exceed the individual tolerability threshold of lactose [7].

This is important, as the exclusion of all dairy products could lead to the development of micronutrient deficiencies. In fact, cow’s milk and dairy products are a major source of calcium, phosphorus, choline, riboflavin, vitamin B12 and vitamin A [18]. In the United States dairy products contribute on average 72% of calcium, 26% of riboflavin, 16% of vitamin A, 20% of vitamin B12, 18% of potassium, 16% of zinc, 15% of magnesium and 19% of high-quality protein [19, 20].

Moreover, two to three daily servings of dairy products are also part of the Mediterranean Diet and the Dietary Approaches to Stop Hypertension (DASH) [19, 21].

Results on nutrient intake among LI subjects show that, compared to tolerant people, they consume lower amounts of calcium, with average intake ranging from 388 to 739 mg a day, below the Recommended Dietary Allowance (RDA) of 1000 mg a day [22–25]. Interestingly, from observational studies it has emerged that the avoidance of dairy products was associated with poor bone health [26, 27], higher blood pressure [28] and an increased risk of diabetes mellitus [29].

In addition, the consumption of yogurt and/or fermented milk plays a fundamental role in the health of the gut microbiota, due to their content in probiotics. A recent systematic review analyzed the potential effect of 8 probiotic strains (*Bifidobacterium longum, Bifidobacterium animalis, Lactobacillus bulgaricus, Lactobacillus reuteri, Lactobacillus acidophilus, Lactobacillus rhamnosus, Saccharomyces boulardii, and Streptococcus thermophilus*) to better explain the rising evidence that probiotic bacteria in fermented and unfermented milk products can be used to improve the clinical symptoms of LI. In conclusion, of the 8 strains studied, *B. animalis* was among the most well-researched and effective strain [30].

The major risk associated with the complete elimination of dairy products from the diet, is that of developing a calcium deficiency and compromising bone health. Therefore, it is fundamental to ensure adequate calcium intake at each stage of life to build and maintain an healthy skeleton, especially in those with LI, who consume less dietary calcium than non-LI individuals [31]. The best sources of dietary calcium include milk, cheese and dairy products, such as broccoli, collards, kale, turnip greens, and fortified soy products. Other foods with less calcium bioavailability are fortified soymilk, sesame seeds, almonds, and red and white beans. Despite this, calcium bioavailability from plant foods can be affected by oxalates and phytates, which are inhibitors of calcium absorption. Another important calcium source is water, in particular, hard water that has high calcium and magnesium levels derived from groundwater [32].

According to the National Medical Association, calcium requirements are the same for males and females during the first 50 years of life (1–3 years: 700 mg Ca/day; 4–8 years and 19–50 years: 1000 mg Ca/day), with the highest recommended intake during the adolescence, when maximal bone growth occurs (9–18 years: 1300 mg Ca/day). These values begin to differ with the onset of menopause: calcium recommended intake for women is increased to 1200 mg Ca/day. This value then evens out as both sexes reach 70 years old, with the recommended daily allowance set at 1200 mg Ca/day, to prevent the development of osteoporosis [33].

To ensure optimal bone mineralization, the American Academy of Pediatrics supports the use of dairy products in children and adolescents [34, 35]. The bone mineral status (BMS) seems to be genetically determined up to 80%, with environmental factors, such as weight, physical exercise, and dietary intake of calcium and vitamin D, affecting it up to 20% [36]. It has been demonstrated that a daily milk consumption of 245 mL (a cup) is associated with increased body height (0.39 cm, 95% CI 0.29 to 0.48) [37]. Baldan and colleagues [38] evaluated the effect of a lactose-free diet on the phalangeal BMS in 102 LI adolescents compared to that of 102 peers on a normal diet. In particular, the time spent on a lactose-free diet (4.8 ± 3.1 years) was inversely correlated to the BMS. The results showed that lactose-free diets did not affect the phalangeal BMS of LI adolescents when they consumed lactose-free cow’s milk; but there was still a significantly lower calcium intake than in the control population.

Moreover, a study by Matlik and colleagues [39] on self-imposed dairy restriction in young girls (10–13 years) showed an approximate 210 mg calcium intake deficit compared with girls that usually consumed dairy products.

In addition to calcium, vitamin D, vitamin A, potassium, zinc, and magnesium in dairy products are also important nutrients in bone formation [8]. In fact, a review performed by Heaney and colleagues [27], consisting of both randomized and observational studies, highlighted the importance of the above mentioned nutrients for bone health. The majority of the analyzed studies concluded that dairy foods are excellent sources for the nutritional requirement for a proper bone status and that it is challenging to reach the recommended calcium intake without the use of dairy products.

## Lactose-free alternatives

In most cases, reducing the consumption of, or avoiding, lactose containing foods and drinks, and replacing them with lactose-free alternatives, is sufficient to control the symptoms of intolerance. There are several alternative foods and drinks options available, both artificial and natural, to replace milk and dairy products, including lactose-free dairy products and plant-based milk food [40] (Table 1).

**Table 1:** Lactose content of common dairy foods

Food	Lactose content (g) per 100 g	Ref.
Milk and derivatives		
Whole milk	4.9	http://www.bda-ieo.it [78]
Skimmed milk	5.3	http://www.bda-ieo.it [78]
Lactose-free milk	0.01–0.1	Churakova et al. [41]
Goat milk	4.7	http://www.bda-ieo.it [78]
Donkey milk	6.1	Malacarne et al. [79]
Cooking cream	3.9	http://www.bda-ieo.it [78]
Sour cream	3.4	https://fdc.nal.usda.gov [80]
Powdered milk	4.2	http://www.bda-ieo.it [78]
Butter	1.1	http://www.bda-ieo.it [78]
Plain yogurt	2.6	http://www.bda-ieo.it [78]
Fruit yogurt	3.2	http://www.bda-ieo.it [78]
Greek yogurt	0.5	http://www.bda-ieo.it [78]
Cultured fermented milk	3.75	https://fdc.nal.usda.gov [80]
Cultured buttermilk	4.5	https://fdc.nal.usda.gov [80]
Fresh cheeses		
Mozzarella cheese	0.7	http://www.bda-ieo.it [78]
Buffalo Mozzarella	0.4	http://www.bda-ieo.it [78]
Ricotta cheese	3.5	http://www.bda-ieo.it [78]
Feta cheese	1.4	http://www.bda-ieo.it [78]
Cottage cheese	3.2	http://www.bda-ieo.it [78]
Lactose-free fresh cheeses	0.01–0.1	Dekker et al. [18]
Hard cheeses		
Cheddar	0.5	http://www.bda-ieo.it [78]
Emmentaler PDO	< 0.1	https://www.emmentaler.ch [81]
Gruyere PDO	< 0.1	https://gruyere.com/ [82]
Fontina PDO	0.8	http://www.bda-ieo.it [78]
Aged cheeses		
Parmigiano Reggiano PDO	< 0.01	Pecorari et al. [49] Coppa et al. [50]
Grana Padano PDO	< 0.01	Monti et al. [51]
Pecorino Romano	< 0.01	Idda et al. [83]
Blue cheeses		
Gorgonzola PDO	< 0.1	https://www.gorgonzola.com/ [52]

These are estimates only; actual lactose content may vary by specific product, brand, or recipe

To meet the dietary calcium and high-quality protein requirements of LI individuals, the global dairy industry has developed lactose-free products using the addition of exogenous lactase, β-galactosidase, which pre-digests the lactose in milk [41]. Lactose-free dairy products allow lactose intolerant subjects to enjoy the taste of dairy without the experience of intestinal symptoms occurring after lactose ingestion. Furthermore, lactose hydrolysis has been reviewed as a sugar reduction option, as the hydrolysis of the lactose in milk enhances the sweetness of the product—the same sweetness intake as adding 2% sugar [42]. Lactose-free dairy is also not expected to have any unusual nutritional effects on the human body when compared to regular dairy products [41]. In particular, no difference was observed in the glycemic response of diabetes patients who consumed lactose or its digestion products, glucose and galactose [43].

Consequently, the broad availability, wide range, and the safety of lactose-free products should encourage consumers to make lactose-free a preferred choice for dairy [18].

### Milk

Lactose-free cow’s milk is available in many countries in different forms. Currently, there are two principal methods to produce this specific milk for intolerant individuals (batch and aseptic methods), and both of them use soluble lactase enzymes [44].

The first method is the batch process, consisting of a pre-hydrolysis process in which neutral lactase is added to the raw milk, and usually incubated for nearly 24 h under moderate stirring to prevent creaming. Furthermore, this process is performed at 4–8 °C to inhibit microbial growth as the milk is not yet sterile. After the incubation, milk is pasteurized, homogenized and packaged [18]. No residual enzyme activity persists in the final product because the enzyme is inactivated throughout the sterilization/pasteurization stage [18]. Enzymes for this process possess a high activity at low temperature and neutral pH and low temperature, so their dosage is relatively high [18].

To reduce the typical doubling of sweetness after lactose hydrolysis, and restored a conventional palatability, ultra and nano-filtration, or chromatography techniques (combined with the hydrolysis of the remaining lactose) are used [18, 45, 46]. The result is excellent quality milk that tastes almost identical to regular milk.

The second method is an aseptic post-hydrolysis process, in which the milk is sterilized using the ultra-high temperature (UHT) procedure. Following this, a sterile lactase preparation is added to the milk just before packaging [18]. The lactose conversion occurs directly in the milk package, and, as UHT milk is often kept in quarantine for almost 3 days, there is sufficient time to achieve the hydrolysis before the product is dispatched to the retailer. Since both temperature and time of incubation are higher in this method, the amount of the enzyme can be much lower when compared to the batch process [18].

### Cheese

There are many lactose-free kinds of cheese available on the market These cheeses are produced by incubating the cheese milk with lactase before renneting. This technique is useful mainly for young and fresh cheeses that contain a significant amount of lactose.

Throughout the cheese making process, the milk is thickened, and the curds (the solid parts) are isolated from the whey (the liquid part where most of the lactose is). Whey is drained off before the cheese is made, so quite a bit of lactose is removed. The curds used to produce hard cheeses have less moisture (whey) than the curds used to make softer cheeses; therefore, soft cheeses possess more lactose than hard ones [18, 47].

More-extra moisture is lost as cheese ages. Moreover, during the aging process, in hard and matured cheeses, lactic acid bacteria consume all the lactose present in the cheese, so no lactase incubation is needed. The longer a cheese has been matured, the less lactose remains in the final product; therefore, the lactose concentration in hard-matured (long-ripened) cheeses is usually very low and can be easily tolerated by most individuals suffering from primary LI.

Parmigiano Reggiano PDO is an example of a hard-matured variety of cheese; it is naturally produced with up to 30% water and 70% nutritious substances, especially protein, calcium and phosphorus [48].

Following the decisions of the Italian Ministry of Health, this is the statement that can be used on the labels of Parmigiano Reggiano packaging: “*Parmigiano Reggiano is naturally lactose free. The absence of lactose is a natural consequence of the traditional Parmigiano Reggiano manufacturing process and it contains less than 0.01* *g/100* *g galactose”* [48]. Indeed, there are natural microbiological conditions for which lactose is absent in Parmigiano Reggiano from the early stages of cheese aging. Scientific investigations validate these statements. In a research study carried out by the Consortium, Pecorari et al. demonstrated that 48 h after production, 0.004 g/100 g of lactose are found in a wheel of Parmigiano Reggiano [49]. Furthermore, Coppa et al., demonstrated that Parmigiano Reggiano is free from lactose and, when analyzing it at different stages of maturation over 1 to 36 months, showed that its lactose content was more than one hundred times lower than the level found by Pecorari and others [50] (Table 1).

Similarly to Parmigiano Reggiano, Grana Padano PDO cheese is naturally lactose free thanks to the characteristics of its production and aging process and has less than 0.01 g/100 g residual galactose content [51] (Table 1).

The Italian Gorgonzola cheese is another naturally lactose free dairy product. The lactose content in Gorgonzola is below the ministerial limit to define a cheese as “naturally lactose-free” (< 0.1 g/100 g). This result is supported by a research conducted by the Consortium for the Protection of Gorgonzola cheese in collaboration with CREA Research Center of Lodi [52] (Table 1).

### Yogurt and other fermented products

Yogurt is a fermented food containing live bacteria, produced from fermented milk. It is nutritionally rich in calcium, riboflavin, vitamin B6, B12, protein and probiotics (live microorganisms which improve the health status of the host by exerting beneficial effects in the gastrointestinal tract [47, 53]).

Most lactose-intolerant people can eat yogurt without exhibiting typical symptoms; moreover, yogurt consumption is suggested as a suitable dietary strategy to reach the recommended daily intake of calcium for LI individuals [54, 55]. In particular, some yogurt culture microorganisms such as *L. delbrueckii* subsp. *bulgaricus* and *S. thermophilus* are able to produce β-galactosidase as part of their lactose utilization pathway and can likely promote lactose digestion in vivo [47, 56].

Despite this, the lactose content is only partially reduced by the original fermentation of yogurt, and most of the lactose survives in the finished product (Table 1). When yogurt is eaten, the live organisms, which contain intracellular β-galactosidase, presumably survive the gastric acidic environment and reach the small intestine, where they are permeabilized by bile acids and release β-galactosidase into the lumen [54, 57]. Thus, any lactose is hydrolyzed by bacterial β-galactosidase, and the glucose and galactose are absorbed across the intestinal epithelium. Nevertheless, many systematic reviews have described that those probiotic bacteria may vary in their ability to improve lactose digestion and reduce maldigestion symptoms [30, 54, 58, 59]. The European Food Safety Authority (EFSA) also examined human clinical studies evaluating the effectiveness of yogurt in improving lactose digestion [60]. According to the expert panel, there is “strong evidence for the biological plausibility of the effect,” and sufficient proof that a cause-effect association existed between yogurt consumption and improved lactose digestion was sufficiently proved to support a health claim for those yogurts containing at least 10^8^ colony-forming units (CFU) per gram [60].

In contrast, cultured buttermilk and sour cream contain similar levels of lactose (Table 1), but, together with other fermented dairy products, are produced using cultures of mesophilic species of Lactococcus and Leuconostoc. These bacteria do not express β-galactosidase and metabolize lactose via a β-galactosidase-independent pathway [54]; thus, lactose is not hydrolyzed, and both of these dairy foods are not well tolerated by lactose intolerant individuals [54].

Furthermore, lactose in yogurt is seen to be better digested due to the decreased transit time of a viscous meal (such as yogurt) compared to a liquid one (such as milk); any extra lactase in the small intestine has more time to digest lactose reducing intolerance symptoms [18, 47, 61–63]. Other factors that appear to improve lactose digestion are eating other foods as part of the same meal and even the choice of particular species of bacteria used in the yogurt-making process [18, 62].

Compared to unflavored yogurts, however, flavored yogurts appear to show slightly decreased lactase activity [18, 61] (Table 1).

Regardless of which of these effects play a significant role in helping LI individuals tolerate fermented milk products, the most reliable solution seems to be the complete enzymatic digestion of lactose in yogurt by incubating the milk with lactase before pasteurization (yogurt from hydrolyzed milk) or adding the lactase together with the culture concurrently with fermentation (co-hydrolysis). In the first option, the amount of added sugar can be decreased since hydrolyzed products taste sweeter because of the higher sweetness of the single monosaccharides; this can result in a product with lower energy [18, 42, 64, 65].

### Non-dairy substitutes

Dairy-free products are principally obtained from plants, such as rice, soy, oats, coconuts, nuts, almonds, cashews, hemp, etc. Nowadays, the consumption of these alternatives has been on the rise, and the food sector has reacted by making these products more available on supermarket shelves [8, 66, 67].

Dairy alternatives usually have a low saturated fat content combined with a lower amount of high-quality protein, minerals and vitamins (calcium, zinc, phosphorus and vitamin B12) compared to cow’s milk. Until recently, fortified soy beverage was the second runner up to dairy milk [8].

When consumed as the main replacement for dairy, vegetable alternatives could have significant health implications, specifically for young children (1–8 years). A Canadian study reported that the consumption of non-dairy substitute drinks was associated with lower childhood height [68]. Only cow’s milk and fortified soy beverages are considered nutritious enough for this age group [69]. Protein, calcium, and vitamin D, other essential nutrients for children’s growth, could also be compromised if relying on solely vegetable-based replacement beverages.

Moreover, the EPIC-Oxford cohort study, with more than 34,000 British people, evidenced that vegans, those individuals that exclude animal products from their diet, have a 30% higher risk of bone fractures when compared to omnivores and vegetarians (consuming dairy products and eggs). This was that was linked to lower calcium intake in vegans compared to omnivores and vegetarians [37]. Further research and long-term studies are required to better understand how cow’s milk (and dairy products) can be safely replaced by plant-based beverages in an individual’s diet to meet the recommended calcium intake [18]. Furthermore, much plant-based food has several added ingredients, including salt, sugar, honey, agave, cane juice, or other sweeteners, adding empty calories to the diet [8].

In conclusion, plant-based diets can be safe for bone health if well planned: increasing the portions of calcium-rich plant foods and using calcium supplements (being careful not to exceed the upper limits).

## The importance of lactose-free labelling

In the last few years, a considerable interest in lactose-free diets as the primary treatment for those suffering from LI has led to a significant growth in the manufacture and sale of lactose-free products and more widespread interest in the health benefits to consumers. However, at the same time, it has become increasingly common within the food industry for lactose and other milk-derived ingredients to be used as additives in non-dairy products. In fact, lactose powder is a common additive in many processed foods, due to its technological properties, mainly enhancing the texture and flavor of many prepared meals. As a result, strict adherence to the lactose-free diet might be difficult for LI patients. Therefore, it is essential that LI individuals constantly check and monitor the labels of all food and drink products they consume [6, 70, 71]. In particular, consumers need to be better educated in the terms used to describe nutritional and food labelling information to completely avoid lactose and allow gut to heal and resolve potential nutritional deficiencies and other associated symptoms.

The first tool for delivering nutrition and health information to consumers is the food label [72]. As reported by the European Food Information Council (EUFIC) [73], the general determinants of the choice of food products include biological (as hunger and palatability), economical (as price and knowledge), physical (e.g., accessibility), social (e.g., family), and psychological (e.g., mood) factors, as well as eating disorders, attitudes, and beliefs.

To protect food allergic/intolerant consumers, European legislation requires the provision of allergen information on food labels. Under European Regulation (EU) No. 1169/2011 [74], the presence of allergens in a food product must be declared in the ingredients list. According to European Regulation (EU) No. 1169/2011 [74], milk and its derivatives—including lactose—should be reported on the label or in the ingredients list.

At the same time, lactose-free product labelling is still controversial. Nowadays, both in European and non-European countries, there is not a universal law regulating the production and commercialization of “delactosed” products, defined as ‘‘lactose-free” or ‘‘low-lactose”, except for infant and follow-on formula in which lactose should be 10 mg/100 kcal [74]. Moreover, also a specific cut-off value establishing the “lactose-free” labelling policy is lacking, as well as the absence of official methods for the determination of lactose in dairy products. The result is the proliferation of many dairy products claiming the absence or reduction of lactose (< 0.01%, < 0.1%, and < 0.5%) differently.

As described by Goodman et al. [75], Front-of-package (FOP) nutritional labels strive to give simple nutritional information in a more accessible position and form compared to the Nutrition Facts table (NFt), which is typically displayed on the back of the packaging [75]. It is even more important when the consumer has special nutritional needs, as in the case of specific allergies and intolerances.

Investigations are confirming that consumers have difficulty understanding and applying the information provided in the NFt, including identifying whether nutrient amounts are “high” or “low” compared to daily guidelines [75].

Goodman et al. have identified several features that increase the effectiveness of FOP systems, including the use of recognizable symbols that are easy to understand combined with simple text descriptors [75].

Food labelling for individuals with Celiac Disease is a good example where a clearly designed symbol has helped to improve the consumer’s awareness of a safer choice. The widespread distribution of foods declared as gluten-free is now possible thanks both to a defined law, that clarified gluten cut-offs levels for gluten-free products, and a visible and easy to recognize symbol supported by an international association for celiac consumer protection.

In Italy, the lactose-intolerant patients’ association, AILI (Associazione Italiana Latto-Intolleranti) has reported that not all consumers are aware of the specific ingredients and foods that could contain lactose, for example buttermilk, anhydrous butter, whey, milk powder, and other variants [76]. For this reason, we believe that there is an urgent need for a specific and universal logo supporting all people suffering from this intolerance. This will help LI individuals to quickly and safely identify and purchase certified lactose-free items when doing their food shopping. AILI has assisted in the creation of the first internationally registered symbol that identifies and certifies lactose-free and milk-free products, named *Lfree*^**®**^.

*Lfree*^**®**^ is a symbol of assurance for lactose intolerant consumers as it is recognized as a European certification mark capable of distinguishing goods and services in respect of specific characteristics, as described in Reg. EU 2015/2424. *Lfree*^**®**^ has been developed using a scientific and technical policy document that identifies specific standards and values required for a product to be certified as lactose-free or milk-free. These standards have been developed specifically for consumers with LI, to clearly and intuitively communicate intuitive and straightforward information using FOP labelling. To date, *Lfree*^**®**^ is the only food brand indicating a clear and immediate message of safety and suitability for a milk and lactose-free diet. In comparison to other food labels for special dietary needs, *Lfree*^**®**^ could be an equivalent symbol for lactose-free products as the crossed grain symbol is for gluten-free products, or the other common certifications that are used for selected subgroups of consumers such as Kosher, Halal or Vegan people [77].

## Conclusions

Improving food labelling is a strategy that could guide consumers to choose safer and healthier products. However, there is a strong need to improve the LI dietary approach and post-diagnosis management. Nutritional education for healthy choices and better understanding of food labels are key factors to improve awareness and avoid lactose-containing products, ensuring adequate nutritional requirements. Considering the frequent use of lactose in many non-dairy foods, the so-called “hidden lactose” (e.g., baked goods, breakfast cereals, drinks, salad dressings, processed meat and powdered meal replacements), it would be helpful to mark safe and suitable products with a well-recognized lactose-free logo. Moreover, improving the development of specific products for LI patients could be an excellent strategy, keeping in mind the current demand, costs, needs of different age groups, and lifestyle changes that a consumer with a food allergy or intolerance will face. In conclusion, food labelling, as well as nutritional and sensory properties of lactose-free products should be maximized to meet consumer’s needs.

## Data Availability

Not applicable.

## Abbreviations

LI: Lactose intolerance
LM: Lactose malabsorption
Ca: Calcium
BMS: Bone mineral status
UHT: Ultra-high temperature
EFSA: European Food Safety Authority
CFU: Colony-forming units
FOP: Front-of-package
NFt: Nutrition facts tables
AILI: Associazione Italiana Latto-Intolleranti
